# Lithium chloride regulates the proliferation of stem-like cells in retinoblastoma cell lines: a potential role for the canonical Wnt signaling pathway

**Published:** 2010-01-13

**Authors:** Amanda K. Silva, Hyun Yi, Sarah H. Hayes, Gail M. Seigel, Abigail S. Hackam

**Affiliations:** 1 Bascom Palmer Eye Institute, University of Miami Miller School of Medicine, Miami, FL; 2 Department of Ophthalmology, SUNY at Buffalo, Buffalo, NY

## Abstract

**Purpose:**

Cancer stem cells are found in many tumor types and are believed to lead to regrowth of tumor mass due to their chemoresistance and self-renewal capacity. We previously demonstrated small subpopulations of cells in retinoblastoma tissue and cell lines that display cancer stem cell-like activities, including expression of stem cell markers, Hoechst dye exclusion, slow cycling, and self-renewal ability. Identifying factors regulating stem cell proliferation will be important for selectively targeting stem cells and controlling tumor growth. Wingless and Int1 (Wnt) signaling is an essential cellular communication pathway that regulates proliferation and differentiation of non-neoplastic stem/progenitor cells in the retina and other tissues, but its role in cancer stem cells in the retinal tumor retinoblastoma is unknown. In this study, we investigated whether the Wnt pathway activator lithium chloride (LiCl) regulates proliferation of retinoblastoma cancer stem-like cells.

**Methods:**

The number of stem-like cells in Weri and Y79 retinoblastoma cell line cultures was measured by 5-bromo-2-deoxyuridine (BrdU) pulse-chase, immunohistochemistry, and quantitative polymerase chain reaction (PCR) for stem cell marker genes. The cell lines were sorted into stem-like and non-stem-like populations by fluorescence-activated cell sorting (FACS), using an antibody against the stem cell marker ATP-binding cassette, subfamily G, member 2 (*ABCG2*). Activated Wnt signaling was measured in the sorted cells by western blotting and immunolocalization of the central mediator β-catenin.

**Results:**

LiCl increased the number of stem-like cells, measured by BrdU retention and elevated expression of the stem cell marker genes *Nanog*, octamer transcription factor 3 and 4 (*Oct3/4*), Musashi 1 (*Msi1*), and *ABCG2*. Sorted *ABCG2*-positive stem-like cells had higher levels of β-catenin than *ABCG2*-negative non-stem cells, suggesting elevated canonical Wnt signaling. Furthermore, stem cell marker gene expression increased after small interfering RNA (siRNA) knock-down of the Wnt inhibitor secreted frizzled-related protein 2 (SFRP2).

**Conclusions:**

These results indicate that the cancer stem-like cell population in retinoblastoma is regulated by canonical Wnt/β-catenin signaling, which identifies the Wnt pathway as a potential mechanism for the control of stem cell renewal and tumor formation in retinoblastoma tumors in vivo.

## Introduction

Solid tumors contain heterogeneous populations of cells with different capacities for proliferation, differentiation, and tumor initiation [1-3]. A population of cells termed cancer stem cells was recently identified at low levels within many types of cancers, including leukemias and cancers of the brain, breast, lung, and prostate [4-7]. These cells have behaviors reminiscent of non-neoplastic stem cells, are undifferentiated yet retain the potential to differentiate, self-renew, and express genes involved in regulating stem cell function. Because cancer stem cells are believed to be chemoresistant and reestablish tumors, they have been proposed as important targets for cancer therapy [8-10].

Retinoblastoma is a pediatric tumor of the retina observed in approximately 1:15,000 live births [11]. A major challenge in the treatment of retinoblastoma is metastatic and secondary tumors that often occur later in life. Current therapies often lead to visual impairment and systemic complications. We previously identified a subpopulation of cells in two retinoblastoma cell lines that express embryonic and neuronal stem cell markers and exhibit label retention and self-renewal, consistent with characteristics of cancer stem cells [12,13]. We also observed stem cell marker genes in retinoblastoma tumor patients, suggesting that the existence of stem-like cells is clinically relevant. Putative cancer stem-like cells were also recently identified in primary retinoblastoma tissue using flow cytometry to detect stem cell and retinal progenitor markers [14].

The cellular signaling pathways that control the proliferation and differentiation of stem-like cells in retinoblastoma are unknown. A promising candidate pathway is the Wingless and Int1 (*Wnt*) signaling pathway, which is implicated in stem cell self-renewal and differentiation in various cancers, and is a well described regulator of non-neoplastic stem cells in many tissues, including retina, skin, and gut [15-17]. Although generally oncogenic [18-20], we and other researchers have observed a tumor suppressive role for Wnt signaling [21-24].

Wnt ligands are secreted glycoproteins that bind to the coreceptors frizzled (FZD) and low density lipoprotein receptor-related protein 5 and 6 (LRP5/6). In the “canonical” Wnt pathway, secreted Wnt ligands bind to their receptors, which activates Disheveled (Dsh) and initiates a series of molecular events that lead to increased β-catenin [25]. Nuclear translocation of β-catenin allows it to bind to T-cell factor/lymphoid enhancer binding factor (TCF/LEF) transcription factors, which activates transcription of Wnt target genes, including genes that control cell division, apoptosis, stem cell phenotype, and metastasis [18,19,26]. In the absence of Wnt ligands, β-catenin levels are maintained at low levels through phosphorylation by the adenomatous polyposis coli (APC)-axin- glycogen synthase kinase 3 beta (GSK3β) protein complex. In many tumors, mutational activation of APC and axin, or constitutive activation of β-catenin, leads to elevated Wnt signaling.

The Wnt signaling pathway regulates viability and maintenance of non-neoplastic stem cells in the retina and ciliary body [27-31]. In chick and mouse retina, canonical Wnt signaling regulates proliferation of retinal stem/progenitor cells and maintains them in an uncommitted state and suppresses neuronal differentiation [30,32,33], whereas in frogs, activating the Wnt pathway promotes progenitor cell differentiation into neurons [28]. These data suggest that the canonical Wnt pathway is a good candidate for regulating proliferation of retinoblastoma stem-like cells. In this study, we characterized the response of stem-like cells in retinoblastoma cell lines to the Wnt pathway activator lithium chloride (LiCl).

## Methods

### Immunostaining

**Figure 1:**
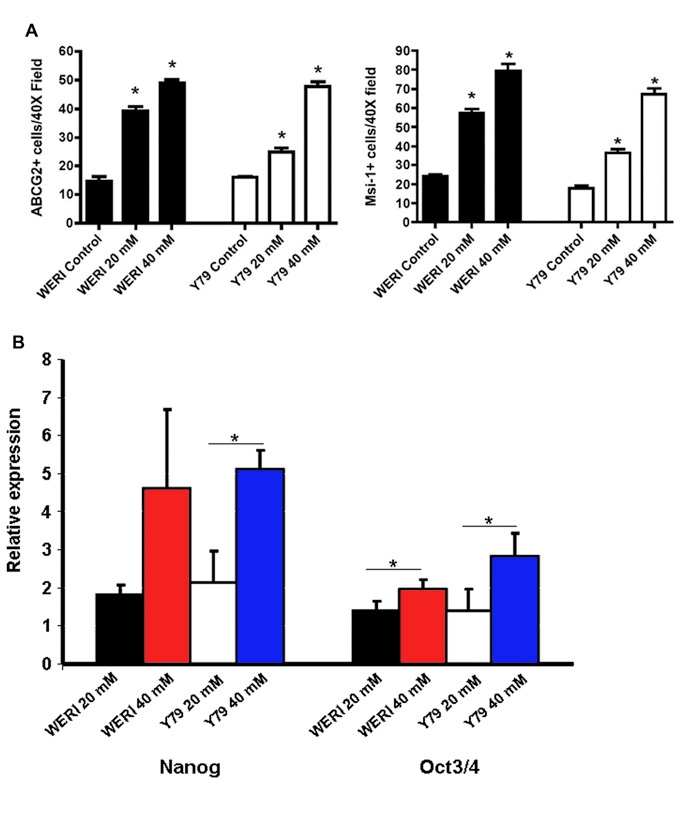
LiCl expands the population of stem-like cells in the Weri and Y79 retinoblastoma cell lines. **A**: LiCl significantly increased the number of ABCG2 and Musashi-1 (Msi-1) immunoreactive cells. Weri and Y79 human retinoblastoma cells were treated with 0, 20, and 40 mM LiCl for 48 h and then immunostained. The number of cells expressing ABCG2 (left) or Musashi-1 (Msi-1; right) were counted. The differences between 20 mM LiCl and control, and 40 mM LiCl and control, were significant at a p-value<0.001 (*). Each experiment was repeated eight times. **B**: LiCl increased the expression of stem cell marker genes Nanog and Oct3/4. QPCR was performed on Weri and Y79 cells treated with 20 mM or 40 mM LiCl. LiCl increased Nanog and Oct3/4 levels, and there was significantly greater expression with 40 mM LiCl treatment compared with 20 mM LiCl at a p-value<0.05, indicated by the asterisk. Each experiment was repeated three times.

**Figure 2:**
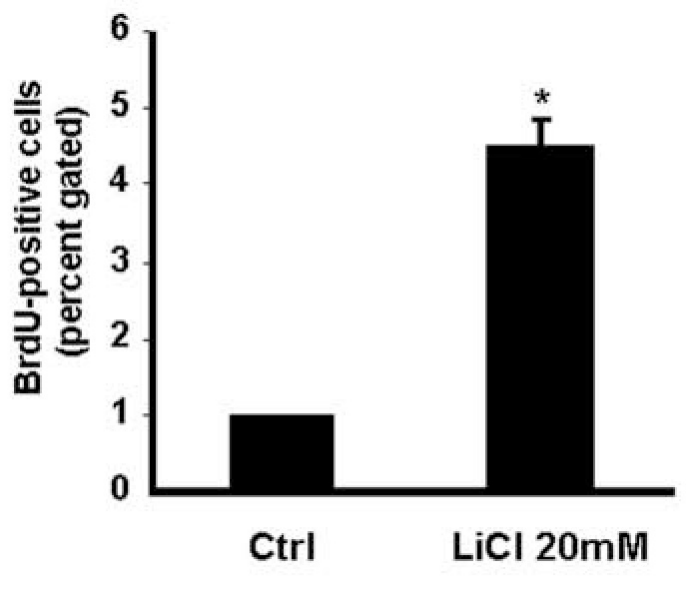
The number of slow cycling cells was increased by LiCl. Y79 cells were incubated with LiCl and pulse-chased with BrdU for a total of 3 weeks. The number of live cells retaining BrdU was measured by flow cytometry. The percent of gated live BrdU+ cells was calculated and normalized to untreated cells and there was significantly greater BrdU+ cells in the LiCl-treated samples at a p-value<0.05, indicated by the asterisk. Each experiment was repeated three times.

**Figure 3:**
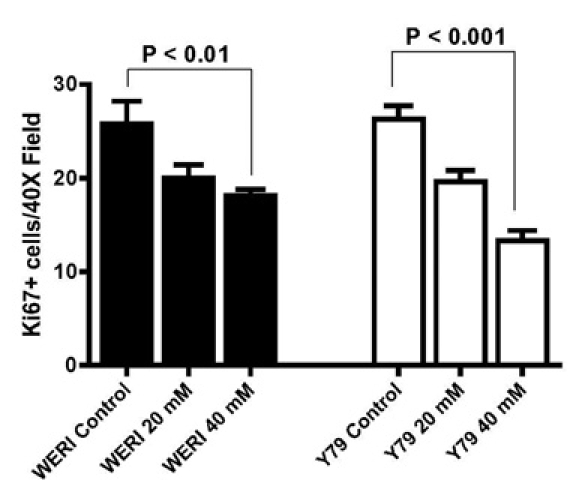
LiCl decreased the proliferative index of the retinoblastoma cell lines. Weri and Y79 retinoblastoma cells were treated with 0, 20, 40 mM LiCl for 48 h and then immunostained. LiCl significantly decreased the number of Ki67 immunoreactive cells (n=8).

The human retinoblastoma cell lines Weri-RB27, a generous gift of Dr. John Ludlow, and Y79, obtained from the American Type Culture Collection (Manassas, VA), were cultured as described previously [13]. The cell lines were treated with 0, 20, or 40 mM LiCl and incubated for 48 h. For the proliferation assays in Figure 1 and Figure 2, control and LiCl-treated cells were fixed for 10 min in cytospin solution, which contained 72% isopropyl alcohol, 19% acetone, 7.6% glycerin. The cells were then centrifuged onto slides for immunocytochemical analysis. The slides were rinsed with phosphate buffer solution (PBS, 137 mM NaCl, 2.7 mM KCl, 10 mM phosphate buffer), blocked with 2% BSA (BSA) in PBS for 10 min then incubated in primary antibody for 1 h. The primary antibody concentrations or dilutions used were 3 μg/ml rabbit anti-Musashi-1 (Msi1, Neuromics, Edina, MN), 1 μg/ml rabbit anti-Ki67 antibody (Ab833, Abcam, Cambridge, MA), 1:500 β-catenin (anti-rabbit from Sigma, St. Louis, MO; anti-mouse from BD Transduction Laboratories, San Jose, CA) and 1:200 dilution of mouse anti- ATP-binding cassette, subfamily G, member 2 (ABCG2; Abcam). After they were rinsed twice in PBS, the slides were incubated in 1 μg/ml goat anti-mouse or anti-rabbit biotinylated secondary antibody (Zymed, San Francisco, CA) for 30–45 min. Following two more washes in PBS, the slides were incubated for 20 min in Vectastain ABC reagent (Vector Laboratories, Temecula, CA), rinsed twice in PBS, and then incubated with Diaminobenzidine (DAB) substrate for 5 min. The slides were washed twice more in PBS, coverslipped with Mowiol anti-fade mounting media (Sigma) and viewed on a Nikon Eclipse E600 microscope. Eight equivalent fields were counted for each concentration of LiCl used. For the β-catenin localization in Figure 3A, the cells were fixed in 4% paraformaldehyde, blocked, and permeabilized using 2% goat serum in 0.3% Triton X-100 in PBS and then incubated in primary antibody. Following several PBS washes, the slides were incubated in 1:600 dilution of secondary antibody (fluorescein isothiocyanate [FITC] or Texas red conjugated, Invitrogen, Carlsbad, CA), washed and then viewed using a fluorescent microscope (Zeiss Axiovert 200, Maple Grove, MN). Images were captured with a digital camera (Axiocam, Zeiss), and photographic and microscopic settings were kept constant for comparisons between antibody and control staining. Eight equivalent fields were counted for per experiment.

### BrdU incorporation

Weri and Y79 cells were plated at a density of 3×10^5^ cells/ml and incubated with 10 μM 5-bromo-2-deoxyuridine (BrdU) for one week with or without LiCl. The cells were then washed three times with media and incubated for an additional two weeks in the absence of BrdU. The cells were collected and washed with 2× salt buffer, which contained 1% BSA and 0.5% NaN_3_ in PBS. Cells were then resuspended in half-salt buffer with 5% fetal bovine serum (FBS), and fixed in ice-cold 70% ethanol. The cells were then denatured with 2 N HCl for 20 min at room temperature and then neutralized with 0.1 M sodium borate for 2 min. The antibody incubations were performed with 1:50 anti-BrdU antibody (BD Transduction Laboratories) for 16 h at 4 C, followed by 1:300 AlexaFlour 488 donkey anti-mouse IgG, a secondary antibody, for 30 min at room temperature. Prior to flow cytometry, propidium iodide and RNase A were incubated with each cell pellet for 20 min, and the cells were then filtered through a 35 micron nylon mesh strainer. Flow cytometry was performed as described [24] using an LSR1 three laser flow cytometer (Becton–Dickinson). The percent of live BrdU-positive cells was calculated, then the background calculated from secondary antibody alone was subtracted, and the samples were normalized to control treatments without LiCl. Unpaired *t*-test or one-way ANOVA and Tukey post-test were used for statistical analyses.

### Cell sorting

The Weri and Y79 cells were pelleted, washed with 2% BSA/PBS and resuspended in 1–2 ml BSA/PBS. The resuspended cells where incubated with anti-ABCG2 antibody (anti-ABCG2-BXP21, Covance) at 4 °C for 20 min, with gentle shaking every 2 min. To control for specificity, the isotype negative control mouse IgG2a antibody (R&D) was used in parallel experiments. The cells were then washed with 10 ml BSA/PBS, resuspended in 1 ml BSA/PBS, and incubated with 5 µl of goat anti-mouse FITC-conjugated, a secondary antibody (Sigma F0257) at 4 °C for 20 min. Following several additional washes, the cells were resuspended in BSA/PBS at 2–3 million cells/ml for sorting by FACS using a FACSAria III apparatus (Becton-Dickinson). The sorted cells were pelleted and then processed for western blotting or RNA analysis, as described previously [24]. For western blot analysis the cells were first lysed in buffer containing proteinase inhibitors (50 mM Tris, pH 7.4, 150 mM NaCl, 1% EDTA, 1% NP-40), and then 20 μg of protein was resolved in 10% SDS–PAGE gels using Tris-Glycine buffer. The proteins were transferred onto polyvinylidene fluoride (PVDF) membranes and probed using the anti-β-catenin antibody described above. For RNA analysis, Trizol reagent (Invitrogen) was used to extract total RNA from the pelleted sorted cells, according to the manufacturer's directions [34]. Briefly, the pellet was homogenized in Trizol, followed by incubation with chloroform and centrifugation to allow phase separation. RNA was precipitated from the aqueous phase using isopropanol, washed with 75% ethanol, dried and then resuspended in water.

### Quantitative PCR

Total RNA was extracted from the retinoblastoma cells using Trizol reagent (Invitrogen), as described above. One microgram of RNA was treated with DNase (Ambion, Austin, TX) and cDNA was synthesized using Thermoscript (Invitrogen). Quantitative PCR was performed using the iCycler thermocycler (BioRad, Hercules, CA) with primers that were specific to the gene of interest and crossed intron-exon boundaries. The primers for *Nanog* [35] and *Oct3/4* are listed in Table 1. Relative transcript levels of each gene were calculated using the delta-delta C_t_ method, using a housekeeping gene as the reference gene.

**Table 1:** Primers used for sybr-green quantitative PCR analysis

**Gene**	**Primer (5′-3′)**	**Product size**
*Nanog*	F: CTAAGAGGTGGCAGAAAAACA	101 bp
	R: CTGGTGGTAGGAAGAGTAAAGG	
*Oct3/4*	F: ACATCAAAGCTCTGCAGAAAGAACTC	126 bp
	R: CTGAATACCTTCCCAAATAGAACCC	

F represents the sense primer; R represents the antisense primer. The size of the amplified product is indicated.

### siRNA transfection

The Silencer small interfering RNA Starter Kit (siRNA; Ambion) was used to reduce secreted frizzled-related protein 2 (SFRP2) expression. siRNAs corresponding to SFRP2 and GAPDH, and a scrambled siRNA for the negative control, were purchased from Ambion. Two independent siRNAs were used against SFRP2: Sfrp2(A) siRNA (ID# s12716; sense 5′-CCA AGA GCA AGA CCA UUU Att-3′, anti-sense 5′- UAA AUG GUC UUG CUC UUG Gtc-3′) and Sfrp2(B) siRNA (ID# s12717; sense 5′-CAU CAA CCG AGA UAC CAA Att-3′, anti-sense 5′-UUU GGU AUC UCG GUU GAU Gta-3′). Weri cells were plated in a six-well plate at 1.5×10^5^ cells/ml and incubated in 2% FBS/RPMI 1640 media before transfection. The siPORT reagent (Ambion) was used for the transfections, according to the manufacturer’s protocol. Transfection efficiency in control transfections was approximately 40%. Briefly, 5 μl of siPORT was diluted in 295 μl serum-free media and incubated for 10 min at room temperature. Next, 12.5 pmol of Sfrp2 siRNA was diluted in serum-free media and mixed with diluted siPORT, incubated for 10 min then added to the cells for 24 h. The cells were collected for RNA extraction, and quantitative PCR was performed as described in the previous section.

## Results

Subpopulations of cells in the retinoblastoma cell lines Weri and Y79 and in primary retinoblastoma tissue were identified with characteristics of stem cells, including marker gene expression, Hoechst dye exclusion, and BrdU label retention [12-14]. To test the hypothesis that the Wnt signaling pathway regulates proliferation of these stem cell-like cells, we induced Wnt signaling in the Weri and Y79 retinoblastoma cell lines by treating with 20 mM and 40 mM LiCl. LiCl activates Wnt signaling by inhibiting GSK3β, leading to β-catenin stabilization and translocation into the nucleus. LiCl was used in this study because it had a greater phenotypic effect on retinoblastoma cell lines than the canonical Wnt ligand Wnt3a [24].

Stem-like cells in Weri and Y79 cell lines were detected by immunoreactivity to the stem cell marker proteins *ABCG2* and *Msi1*. As shown in Figure 1A, the baseline number of cells expressing these proteins in untreated controls was 10–20 cells per 40× field. LiCl treatment significantly increased the number of *ABCG2*-positive cells up to 50 cells per field in both Weri and Y79 cell lines. LiCl also significantly increased the number of cells that expressed the stem cell marker protein Msi1 to approximately 80 cells per field.

The expression of two additional stem cell marker genes, *Nanog* and *Oct3/4*, was analyzed. Quantitative PCR was used because of high background in the immunostaining for these two proteins. LiCl increased *Nanog* and *Oct3/4* expression in the cell lines (Figure 1B). All four of the genes in Figure 1 had significantly greater expression with 40 mM LiCl compared with 20 mM LiCl. Therefore, LiCl increased the expression and immunoreactivity of multiple stem cell genes, indicating that LiCl expands the population of stem-like cells in the Weri and Y79 retinoblastoma cell lines.

We next used a functional assay to quantify the effect of LiCl on proliferation of the stem-like cells. A commonly used functional marker of cancer stem cells is slow cycling [13], which may cause chemoresistance by allowing time for the cells to repair damaged DNA [8]. Slow cycling can be detected by retention of BrdU in a pulse-chase experiment. Y79 cells were used in this experiment because they had higher expression of the stem cell marker genes than Weri cells in Figure 1B, predicting a greater effect with BrdU. To determine whether LiCl alters the number of cells that retain BrdU, we incubated Y79 cells with LiCl and BrdU and used flow cytometry to quantitate the number of live cells that were BrdU-positive. As shown in Figure 2, LiCl treatment significantly increased the number of slow cycling cells by approximately fourfold, indicating an expansion of the number of stem-like cells in the culture.

Reduced proliferation rate can also be measured by detection of the proliferation marker Ki67. We quantified the effect of LiCl on the number of cells that were immunoreactive for the Ki67 protein by comparing to untreated cultures. LiCl treatment of Weri and Y79 cells significantly decreased the number of Ki67-positive cells to 10 cells/field in LiCl-treated cultures from 35 cells/field in control cultures (Figure 3).

Wnt signaling maintains the stem phenotype of non-neoplastic stem cells in the mammalian retina [30,32,33]. Therefore, we next asked whether the stem-like cells in the retinoblastoma cell lines have higher levels of endogenous Wnt signaling than the nonstem cells. To address this question, Wnt signaling levels were measured using western blotting for β-catenin. Increased β-catenin levels is a well established marker of canonical Wnt pathway activation. Our previous study in Weri and Y79 cell lines demonstrated low β-catenin levels that could be induced by exogenous Wnt pathway activators, indicating that the canonical Wnt pathway was downregulated but that it is intact and functional [24].

**Figure 4:**
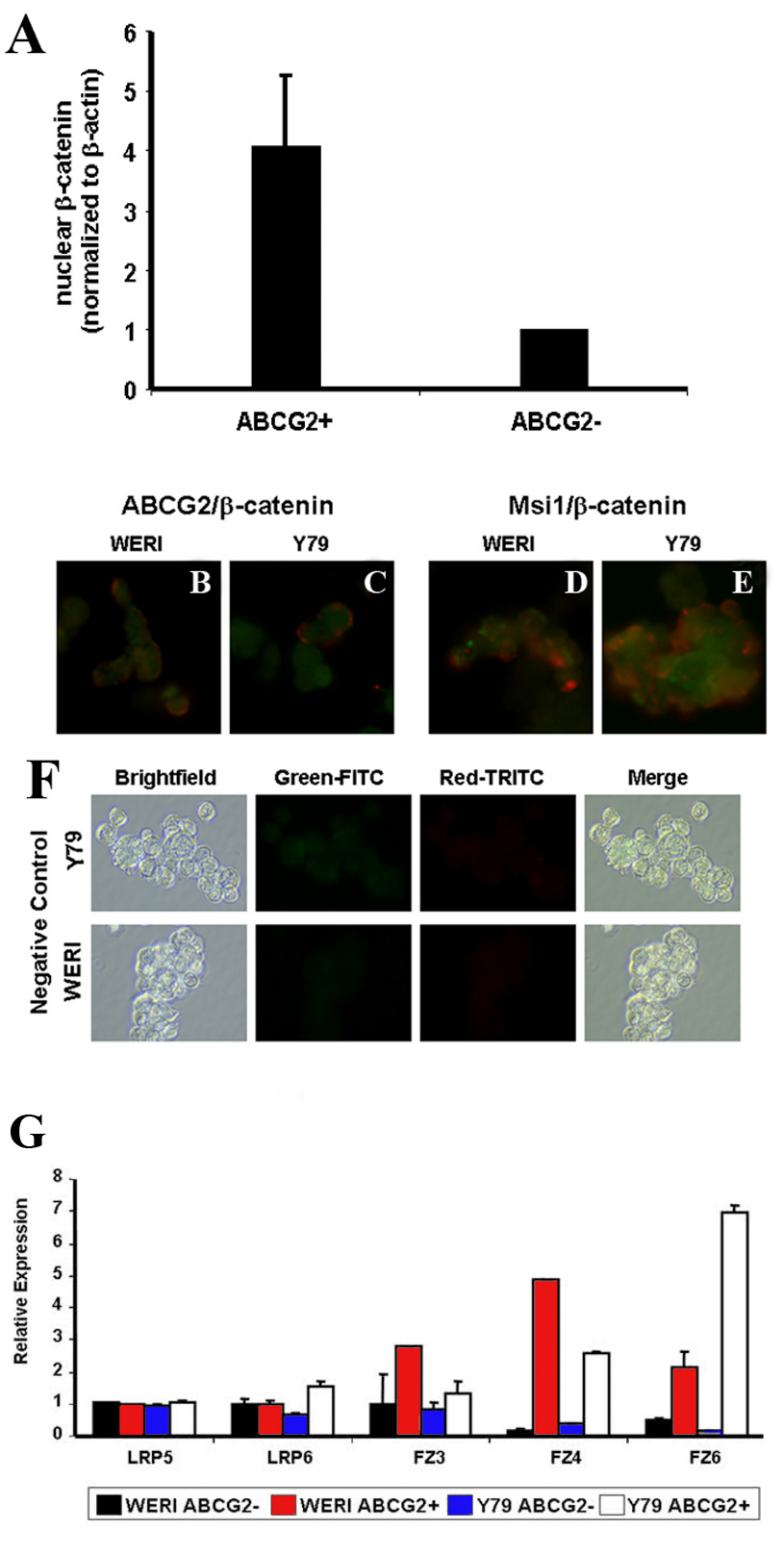
Elevated canonical Wnt signaling in flow-sorted retinoblastoma cancer stem-like cells. **A**: Weri cells were sorted by flow cytometry using an antibody against the stem cell marker ABCG2. RNA and protein were then extracted from the ABCG2-positive and ABCG2-negative cells. Western blotting on sorted ABCG2+ stem cells showed higher β-catenin levels, indicating higher canonical Wnt signaling in the stem cells (n=3, p=0.048). β-catenin levels were normalized to β-actin, and are expressed as a ratio of the respective ABCG2-negative cells. **B-F**: Representative images showing the immunoreactivity of the stem cell markers ABCG2 and Msi1, and the canonical Wnt signaling mediator β-catenin in the retinoblastoma cell lines Weri and Y79. In the top right panels, ABCG2 immunoreactivity is red and β-catenin is green. In the top left panels, Msi1 immunoreactivity is green and β-catenin is red. The bottom panels show the negative controls, in which Weri and Y79 cells were treated with control rabbit IgG and FITC or TRITC secondary antibodies. The adjacent bright-field images are also shown. All images were taken at 40x. **G**: QPCR on canonical Wnt signaling receptors demonstrated higher expression of the receptors LRP6, Fz3, Fz4, and Fz6 in the in ABCG2-positive (cancer stem-like cells) than ABCG2-negative (non-stem-like cells). Data are from two independent experiments; each experiment had three replicates.

The stem-like cells in Weri and Y79 cell lines represent approximately 4% of the total cell population [13]. Therefore, to compare Wnt signaling in stem-like cells with nonstem cells, we first sorted the cells using flow cytometry with the stem cell marker protein ABCG2. Western blotting for β-catenin on the ABCG2-positive stem cells from Weri cultures demonstrated 4.1-fold (SD±1.2) higher β-catenin levels than the ABCG2-negative cells (p<0.05, n=3), indicating greater canonical Wnt signaling in the stem-like cells (Figure 4A). Examples of double-staining of ABCG2 and β-catenin, and Msi1 and β-catenin, are shown in Figure 4B-E. Detection of the stem cell markers were rare events in unsorted cells in relation to the entire culture. Stem cell-immunoreactive cells tended to appear in clusters, possibly due to mother-daughter cell proximity.

To identify a potential mechanism for upregulated β-catenin levels in ABCG2-positive stem cells, we compared levels of canonical Wnt signaling receptors using quantitative PCR analysis. As shown in Figure 4G, there was higher expression of the receptors *LRP6*, *Frizzled 3* (*Fz3*), *Fz4,* and *Fz6* in the ABCG2-positive stem-like cells than in ABCG2-negative nonstem cells. Interestingly, there were also differences in receptor expression between the cell lines.

**Figure 5:**
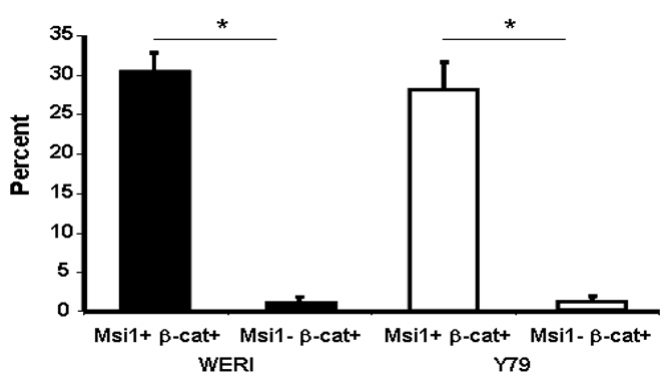
Cancer stem-like cells have active Wnt signaling in the retinoblastoma cell lines. The percent of co-localized Msi1 and nuclear β-catenin was quantified in the Weri and Y79 cell lines. Immunohistochemistry demonstrated that tumor stem cells (Msi1+) were more often positive for nuclear β-catenin than non-stem cells (Msi-), indicating active Wnt signaling. The difference in nuclear β-catenin was statistically significant at p<0.001 (*). The experiment was repeated five times.

We next used immunodetection to quantify the number of stem-like cells with Wnt signaling using Msi1 as a stem cell marker (Figure 5). We found that 30.6% (SD±2.2) of Msi1-positive cells contained nuclear β-catenin in the Weri cell line, and 28.2% (SD±3.4) cells contained nuclear in the Y79 cell line. In contrast, veryy few Msi1-negative cells in the Weri (1.31%. SD±0.55) and Y79 (1.21%, SD±0.49) contained nuclear β-catenin in both cell lines (p<0.001 compared with Msi1-positive cells, n=5), indicating that retinoblastoma cells with active Wnt signaling were more likely to be stem cells.

**Figure 6:**
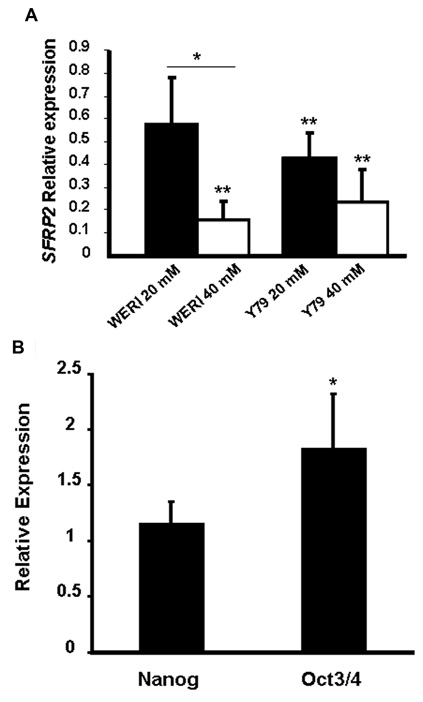
The Wnt inhibitor SFRP2 regulates cancer stem-like cells. **A**: The Wnt inhibitor SFRP2 was decreased in Weri and Y79 cells by addition of LiCl, as measured by QPCR on unsorted cells. The difference between 20 mM and 40 mM LiCl was statistically significant at p<0.05 (*) and the difference between treated and untreated was statistically significant at p<0.01 (**). The experiment was repeated four times. **B**: Reducing SFRP2 levels using siRNA (average % reduction was 63% relative to scrambled siRNA) increased stem cell marker gene expression. The increase in *Oct3/4* expression was statistically significant at p=0.05 (*). The experiment was repeated four times.

The secreted Wnt inhibitor SFRP2 was recently shown to be a differentiation-promoting gene in retinal stem/progenitor cells in rat [36]. Reduction of SFRP2 levels by activating the Notch signaling pathway leads to disinhibition of Wnt signaling, increased proliferation of stem/progenitor cells and decreased expression of proneural genes [36]. Therefore, SFRP2 is an attractive candidate molecule for mediating stem cell proliferation by LiCl/Wnt signaling in retinoblastoma cell lines. Quantitative PCR on LiCl-treated cell lines demonstrated that *SFRP2* was reduced by up to sixfold in Weri and fourfold in Y79 cells (Figure 6A).

To determine whether SFRP2 regulates retinoblastoma stem cells, we experimentally reduced *SFRP2* levels, using siRNA, and employed expression of *Nanog* and *Oct3/4* as markers of the stem cell phenotype. Weri cells were used in this experiment because they had a higher transfection efficiency than Y79 cells. As shown in Figure 6B, reducing *SFRP2* levels by siRNA transfection (average reduction was 63% relative to scrambled siRNA, n=4) resulted in 80% increase in *Oct3/4* expression (range: 30%–130% increase, n=4), similar to the effect of LiCl (see Figure 1B). Expression of *Nanog* was increased by approximately 20% by the SFRP2 siRNA. Total β-catenin levels were used as a measure of canonical Wnt signaling, as shown previously in the Weri and Y79 cell lines [24]. Western blotting of β-catenin in cells transfected with siRNA targeting SFRP2 showed a 38% increase (data not shown). The small increase in β-catenin levels may be due to the baseline Wnt signaling levels being low in these cell lines [24] such that disinhibition by suppressing SFRP2 does not result in substantial increases.

In conclusion, the number of cells with a stem-like phenotype in retinoblastoma cell lines was increased by treatment with LiCl and by reducing the expression of the canonical Wnt pathway inhibitor SFRP2. These data suggest that canonical Wnt signaling is an important regulator of retinoblastoma stem-like cells in vivo.

## Discussion

Retinoblastoma is a retinal tumor that is proposed to derive from pluripotent progenitor cells [37]. Wnt signaling is a likely candidate pathway for playing a regulatory role in stem-like cells in retinoblastoma tumors because of its function in proliferation and differentiation of non-neoplastic stem/progenitor cells in the retina. The ciliary marginal zone (CMZ) at the periphery of the retina in fish and amphibians contains progenitor cells that have stem cell properties, including self-renewal and multipotency [38]. In birds, the CMZ stem/progenitor cells are also proliferative but have more limited differentiation capacity. In the analogous region in mammals, the pigmented ciliary margin, stem/progenitor cells are believed to be quiescent in vivo and act like stem cells when cultured in vitro [39,40]. Activation of the canonical Wnt pathway in chick and mouse retinal stem/progenitor cells promotes proliferation and inhibits neuronal differentiation [30,32,33], whereas Wnt signaling promotes neurogenesis in *Xenopus* retina CMZ [28]. Wnt signaling also increases the number of microspheres generated from the mammalian pigmented ciliary margin [36,41], suggesting that Wnt signaling also maintains the stem cell population in mammals. Furthermore, Wnt signaling was proposed to activate stem cell properties in Muller glia and promote their proliferation during retinal injury [36,42]. Our data suggest that the postembryonic role of Wnt in neoplastic stem cells may recapitulate its embryonic function as a mitogenic regulator of stem/progenitor cells during retinal development.

Wnt signaling has been implicated as a stem cell growth factor in several cancer types [16,43,44]. Mutations that activate the Wnt pathway are suggested to cause constitutive renewal and expansion of the stem cell pool, or to confer a stem cell phenotype (renewal) to the progenitor cell pool [16,44,45]. Future studies will investigate whether the increased numbers of stem-like cells that we observed in the retinoblastoma cell lines are due to proliferation of existing stem cells and/or conversion of nonstem cells to stem-like cells. Another interesting observation was the difference in receptor levels and stem cell marker genes between the cell lines, with Y79 cells having a greater response. An intriguing possibility is that the different responses may correlate with differential metastatic potential of these cell lines [46].

In the present study, we used cell lines to perform basic characterizations of the response of stem-like cells to LiCl, based on previous findings of low but consistent levels of stem-like cells in the human retinoblastoma tissue and cell lines [13,14]. Cell lines offer the convenience of large numbers of cells to analyze: they do not require vascularization and specific systemic requirements of solid tumors in vivo, and are unlikely to be contaminated by non-neoplastic stem cells [47]. Because the cell lines are maintained in culture for long periods, they are unlikely to completely represent the tumor phenotype. However, cell lines are useful for generating questions that can subsequently be addressed in vivo.

LiCl was used in this study because it had a greater effect on cell cycle arrest and viability in the retinoblastoma cell lines than the canonical Wnt ligand Wnt3a [24]. Although generally used to activate canonical Wnt signaling by inhibiting GSK3β, LiCl can also activate non-Wnt pathways [48]. Furthermore, GSK3β itself mediates several signaling pathways [49], and regulates the hematopoietic stem cell repopulation through the Wnt, Notch, and Hedgehog pathways [50]. Unfortunately, using a Wnt ligand to activate Wnt signaling produced inconsistent results, and we were unable to compare with the response to LiCl. Our findings of increased nuclear β-catenin and elevated Wnt ligands in the stem-like cells, and the results from knocking down SFRP2 to activate the Wnt pathway, suggest that Wnt signaling is involved in regulating the number of stem-like cells in the retinoblastoma cell lines. The possibility remains that LiCl also acts through additional molecular pathways. Identifying other LiCl-regulated signaling pathways that control survival and proliferation of retinoblastoma stem-like cells, and elucidating pathways that cross-talk with the Wnt pathway, will be the focus of future studies.

SFRP2 antagonizes the canonical Wnt/β-catenin pathway by acting as a decoy receptor for Wnt ligands and prevents their interaction with the co-receptor Frizzled. Our results suggest that SFRP2 is downregulated by LiCl, and its expression may antagonize maintenance of the stem-like cell population. SFRP2 is an attractive candidate molecule for controlling stem cell proliferation in retinoblastoma tumors in vivo. SFRP2 is expressed during retinal development [51] and regulates differentiation in non-neoplastic stem cells in the retina [52], cardiomyocytes [53], and the pluripotent mouse embryonal carcinoma stem cell line P19CL6 [53]. Interestingly, SFRP2 increases as retinal stem/progenitor cells differentiate into ganglion cells, which was associated with decreased Wnt signaling [52]. In contrast, in the stem cell line P19CL6, SFRP2 maintained the cells in an undifferentiated state [53]. SFRP2 is also a tumor suppressor in many cancer types, including colorectal, cervical, breast, and ovarian, and is inactivated by hypermethylation of its promoter [54].

The response to Wnt signaling activators in a given tissue is dependent on the intersection of other signaling pathways, and can vary in the same cell population over time. Our data suggest that Wnt signaling has two mechanisms of action in retinoblastoma, and emphasizes the heterogeneous nature of the response of tumors and tumor cell lines. In the current study, we demonstrated that the Wnt signaling activator LiCl increased the number of stem-like cells. In our previous work, we showed that Wnt signaling (induced by LiCl and Wnt3a ligand) has tumor suppressor properties in retinoblastoma and induces cell cycle arrest in retinoblastoma cell lines [24]. Therefore, these data suggest that the growth of the bulk of the tumor is halted by Wnt signaling whereas the stem-like cells expand. Future work will investigate the basis of the differential response to Wnt signaling. A therapy that decreases the bulk of the retinoblastoma tumor using Wnt activator proteins would need to be used in combination with inhibitors of the Wnt pathway that are specifically targeted to the stem cell population, potentially by using ABCG2 or other cancer stem cell marker genes.
